# Supplementary material for: Artificial Intelligence Reveals Distinct Prognostic Subgroups of Muscle-Invasive Bladder Cancer on Histology Images
Source: Cancers (Basel). 2023 Oct 16;15(20):4998. doi: 10.3390/cancers15204998 (PMC10605516; doi:10.3390/cancers15204998)
Supplement: Supplementary file 1 [file cancers-15-04998-s001.zip › cancers-2573860-supplementary.pdf]

Supplementary File S1

Supplementary Tables for "Artificial Intelligence Reveals Distinct Prognostic Subgroups of Muscle-Invasive Bladder Cancer on Histology Images"

Table of Contents

SIGNATURE LIST 3

MICRORNA CLUSTER 6

MUTATION IN TSC1 6

MRNA CLUSTER 7

MUTATION IN FGFR3 7

LNCRNA CLUSTER 7

MUTATION IN ERBB3 7

MUTATION IN FAT1 7

MUTATION IN PIK3CA 8

MUTATION IN KANSL1 8

MUTATION IN TMCO4 9

MUTATION IN KDM6A 9

MUTATION IN METTL3 9

SQUAMOUS PATHOLOGY 9

MUTATION IN PSIP1 9

MUTATION IN ZNF773 9

HYPOMETHYLATION CLUSTER 9

MUTATION IN GNA13 10

Signature list

No.	Signature
1	Pathology classification
2	Pathology abbreviated
3	Inflammatory Infiltrate Response
4	Squamous pathology
5	Neuroendocrine pathology
6	Plasmacytoid pathology
7	NOS pathology
8	mRNA cluster
9	Hypermethylation cluster
10	Hypomethylation cluster
11	microRNA cluster
12	lncRNA cluster
13	RPPA cluster
14	Mutation process cluster
15	SMG SCNA cluster
16	mutation in TP53
17	mutation in RB1
18	mutation in RHOB
19	mutation in PIK3CA
20	mutation in KDM6A
21	mutation in TSC1
22	mutation in ELF3
23	mutation in KMT2D
24	mutation in CREBBP
25	mutation in CDKN1A
26	mutation in EP300
27	mutation in ZFP36L1
28	mutation in ARID1A
29	mutation in STAG2
30	mutation in CDKN2A
31	mutation in HRAS
32	mutation in KRAS
33	mutation in FBXW7
34	mutation in ERCC2
35	mutation in ASXL2
36	mutation in RHOA
37	mutation in KMT2A

38	mutation in FGFR3
39	mutation in NFE2L2
40	mutation in KMT2C
41	mutation in PSIP1
42	mutation in KANSL1
43	mutation in C3orf70
44	mutation in FAT1
45	mutation in SPTAN1
46	mutation in RXRA
47	mutation in ZBTB7B
48	mutation in PTEN
49	mutation in ATM
50	mutation in KLF5
51	mutation in PARD3
52	mutation in CUL1
53	mutation in NRAS
54	mutation in SF3B1
55	mutation in GNA13
56	mutation in RBM10
57	mutation in ACTB
58	mutation in MBD1
59	mutation in CASP8
60	mutation in HIST1H3B
61	mutation in TAF11
62	mutation in ERBB2
63	mutation in NUP93
64	mutation in SF1
65	mutation in ERBB3
66	mutation in METTL3
67	mutation in SPN
68	mutation in MB21D2
69	mutation in SSH3
70	mutation in USP28
71	mutation in ASXL1
72	mutation in TMCO4
73	mutation in HES1
74	mutation in ZNF773
75	total number Single Nucleotide Variants
76	total number Indels

77	NMF based count C T_CpG mutations
78	NMF based count ERCC2 mutations
79	NMF based count APOBEC b mutations
80	NMF based count APOBEC a mutations
81	NMF based fraction C T_CpG mutations
82	NMF based fraction ERCC2 mutations
83	NMF based fraction APOBEC b mutations
84	NMF based fraction APOBEC a mutations
85	APOBEC induced mutation load PMACD
86	APOBEC mutation load tertile
87	ABSOLUTE call status
88	Tumor purity from ABSOLUTE
89	Tumor ploidy from ABSOLUTE
90	Genome doublings from ABSOLUTE
91	Cancer DNA fraction from ABSOLUTE
92	Subclonal genome fraction from ABSOLUTE
93	focal amplification in E2F3.SOX4
94	focal amplification in PVRL4
95	focal amplification in YWHAZ
96	focal amplification in CCND1
97	focal amplification in PPARG
98	focal amplification in MDM2
99	focal amplification in GATA3
100	focal amplification in ZNF703
101	focal amplification in CCNE1
102	focal amplification in MYCL1
103	focal amplification in BCL2L1
104	focal amplification in TERT
105	focal amplification in ERBB2
106	focal amplification in MCL1
107	focal amplification in EGFR
108	focal amplification in AHR
109	focal amplification in JAK2
110	focal amplification in FGFR3
111	focal amplification in KRAS
112	focal deletion in CDKN2A
113	focal deletion in RB1
114	focal deletion in PDE4D
115	focal deletion in CCSER1

116	focal deletion in CREBBP
117	focal deletion in WWOX
118	focal deletion in LRP1B
119	focal deletion in PTEN
120	focal deletion in ARID1A
121	focal deletion in NCOR1
122	focal deletion in RAD51B
123	focal deletion in FHIT
124	focal deletion in PTPRD
125	focal deletion in KDM6A
126	Neoantigen load
127	HLA mutation
128	Fusion in FGFR3
129	Fusion in PPARG
130	Fusion in PTPN13
131	Fusion in RHOA
132	Fusion in TNFRSF21
133	Fusion in ASIP

microRNA cluster

Subtype	OR	CI 95% (lower)	CI 95% (upper)	Fisher test	Rank	B-H critical value	Significance
3	0.4891673	0.30949848	0.7675367	0.001237183	1	0.04	*
1	1.7112831	1.00492709	2.9496735	0.042928036	2	0.08	*
ND	0.3243834	0.03166642	1.8416907	0.173849383	3	0.12	nc
2	1.3007998	0.83483947	2.0323424	0.238318513	4	0.16	nc
4	1.2302360	0.73087070	2.0801078	0.453396719	5	0.20	nc

mutation in TSC1

Status	OR	CI 95% (lower)	CI 95% (upper)	Fisher test	Rank	B-H critical value	Significance
WT	2.811912	1.3106471	6.4519114	0.004093058	1	0.1	*
MUT	0.355630	0.1549928	0.7629819	0.004093058	2	0.2	*

mRNA cluster

Subtype	OR	CI 95% (lower)	CI 95% (upper)	Fisher test	Rank	B-H critical value	Significance
Luminal_papillary	0.5503452	0.356269439	0.8460994	0.005136399	1	0.03333333	*
Basal_squamous	1.7430838	1.129631392	2.7027458	0.009110960	2	0.06666667	*
Neuronal	2.1968868	0.919444565	5.6586275	0.076581201	3	0.10000000	*
ND	0.3276581	0.006201882	4.1192275	0.370745811	4	0.13333333	nc
Luminal_infiltrated	0.8143632	0.477874711	1.3820029	0.447574880	5	0.16666667	nc
Luminal	1.0850770	0.426822692	2.7858274	1.000000000	6	0.20000000	nc

mutation in FGFR3

Status	OR	CI 95% (lower)	CI 95% (upper)	Fisher test	Rank	B-H critical value	Significance
WT	2.0577819	1.1532480	3.7485316	0.01016707	1	0.1	*
MUT	0.4859601	0.2667711	0.8671162	0.01016707	2	0.2	*

lncRNA cluster

Subtype	OR	CI 95% (lower)	CI 95% (upper)	Fisher test	Rank	B-H critical value	Significance
3	0.4837417	0.2739837	0.8391926	0.006654241	1	0.04	*
4	1.7092592	1.1165815	2.6275041	0.010342543	2	0.08	*
2	0.7905216	0.4981848	1.2510181	0.318221280	3	0.12	nc
1	1.2093942	0.7041715	2.0869989	0.517195051	4	0.16	nc
ND	0.9901708	0.1817876	5.3932136	1.000000000	5	0.20	nc

mutation in ERBB3

Status	OR	CI 95% (lower)	CI 95% (upper)	Fisher test	Rank	B-H critical value	Significance
WT	2.1902027	1.0990852	4.5361162	0.01792408	1	0.1	*
MUT	0.4565787	0.2204529	0.9098476	0.01792408	2	0.2	*

mutation in FAT1

Status	OR	CI 95% (lower)	CI 95% (upper)	Fisher test	Rank	B-H critical value	Significance
WT	0.5339163	0.2977397	0.9413875	0.02328952	1	0.1	*
MUT	1.8729526	1.0622618	3.3586387	0.02328952	2	0.2	*

mutation in PIK3CA

Status	OR	CI 95% (lower)	CI 95% (upper)	Fisher test	Rank	B-H critical value	Significance
WT	0.6035109	0.3680987	0.9818816	0.03596012	1	0.1	*
MUT	1.6569708	1.0184528	2.7166623	0.03596012	2	0.2	*

mutation in KANSL1

Status	OR	CI 95% (lower)	CI 95% (upper)	Fisher test	Rank	B-H critical value	Significance
WT	2.0690776	0.9841384	4.546840	0.04184239	1	0.1	*
MUT	0.4833072	0.2199330	1.016117	0.04184239	2	0.2	*

mutation in TMCO4

Status	OR	CI 95% (lower)	CI 95% (upper)	Fisher test	Rank	B-H critical value	Significance
WT	4.6910592	0.95414097	45.155757	0.03543734	1	0.1	*
MUT	0.2131715	0.02214557	1.048063	0.03543734	2	0.2	*

mutation in KDM6A

Status	OR	CI 95% (lower)	CI 95% (upper)	Fisher test	Rank	B-H critical value	Significance
WT	1.5842404	1.011445	2.4934236	0.03981648	1	0.1	*
MUT	0.6312173	0.401055	0.9886841	0.03981648	2	0.2	*

mutation in METTL3

Status	OR	CI 95% (lower)	CI 95% (upper)	Fisher test	Rank	B-H critical value	Significance
WT	3.477753	0.87812271	19.952959	0.05269398	1	0.1	*
MUT	0.287542	0.05011788	1.138793	0.05269398	2	0.2	*

Squamous pathology

Existence status	OR	CI 95% (lower)	CI 95% (upper)	Fisher test	Rank	B-H critical value	Significance
Absent	0.5472881	0.2776081	1.051119	0.06712539	1	0.1	*
Present	1.8271912	0.9513669	3.602200	0.06712539	2	0.2	*

mutation in PSIP1

Status	OR	CI 95% (lower)	CI 95% (upper)	Fisher test	Rank	B-H critical value	Significance
WT	2.450398	0.8629208	7.944669	0.07018782	1	0.1	*
MUT	0.408097	0.1258706	1.158855	0.07018782	2	0.2	*

mutation in ZNF773

Status	OR	CI 95% (lower)	CI 95% (upper)	Fisher test	Rank	B-H critical value	Significance
WT	3.1145202	0.76275902	18.14406	0.08645945	1	0.1	*
MUT	0.3210767	0.05511445	1.31103	0.08645945	2	0.2	*

Hypomethylation cluster

Subtype	OR	CI 95% (lower)	CI 95% (upper)	Fisher test	Rank	B-H critical value	Significance
4	0.4449481	0.1901593	0.9818698	0.03231970	1	0.04	*
2	1.6759930	0.9604561	2.9648401	0.06443676	2	0.08	*
3	0.8040387	0.4627858	1.3899148	0.43307045	3	0.12	nc
1	1.1131818	0.7338594	1.6899079	0.61356526	4	0.16	nc
5	0.9581662	0.5717707	1.6047348	0.90168307	5	0.20	nc

mutation in GNA13

Status	OR	CI 95% (lower)	CI 95% (upper)	Fisher test	Rank	B-H critical value	Significance
WT	2.5065931	0.8034681	9.257219	0.08828155	1	0.1	*
MUT	0.3989479	0.1080238	1.244604	0.08828155	2	0.2	*
